# Alternatives to Conventional Topical Dosage Forms for Targeted Skin Penetration of Diclofenac Sodium

**DOI:** 10.3390/ijms25137432

**Published:** 2024-07-06

**Authors:** Benjamin Gavinet, Séverine Sigurani, Christine Garcia, Alicia Roso

**Affiliations:** Research & Innovation, Seppic, 127 Chemin de la Poudrerie, 81100 Castres, France; benjamin.gavinet@airliquide.com (B.G.); alicia.roso@airliquide.com (A.R.)

**Keywords:** cream–gel, gel-in-oil emulsion, topical formulation, skin penetration, diclofenac, in vitro permeation test

## Abstract

Skin penetration of an active pharmaceutical ingredient is key to developing topical drugs. This penetration can be adjusted for greater efficacy and/or safety through the selection of dosage form. Two emerging dosage forms, cream–gel and gel-in-oil emulsion, were tested for their ability to deliver diclofenac into the skin, with the target of maximising skin retention while limiting systemic exposure. Prototypes with varying amounts of solvents and emollients were formulated and evaluated by in vitro penetration testing on human skin. Cream–gel formulas showed better skin penetration than the emulgel benchmark drug even without added solvent, while gel-in-oil emulsions resulted in reduced diffusion of the active into the receptor fluid. Adding propylene glycol and diethylene glycol monoethyl ether as penetration enhancers resulted in different diclofenac penetration profiles depending on the dosage form and whether they were added to the disperse or continuous phase. Rheological characterisation of the prototypes revealed similar profiles of cream–gel and emulgel benchmark, whereas gel-in-oil emulsion demonstrated flow characteristics suitable for massaging product into the skin. This study underlined the potential of cream–gel and gel-in-oil emulsions for adjusting active penetration into the skin, broadening the range of choices available to topical formulation scientists.

## 1. Introduction

Targeted skin delivery of the active pharmaceutical ingredient (API) contained in a drug product is key to maximising its efficacy, while also reducing side-effects arising from unnecessary exposure. Skin diffusion is intrinsically linked to the physicochemical properties of the active, but also to the excipients used and the overall dosage form [1].

Recent studies have highlighted interest in new dosage forms for their impact on skin penetration of active pharmaceutical ingredients as compared to conventional semi-solid dosage forms like gels, emulgels, creams, or ointments. Microneedles can be one example, with their potential to by-pass the stratum corneum, the main skin barrier, and deliver therapeutic compounds directly into the epidermis or dermis [2]. Nanoemulsions and nanocarriers (nanosize vesicles, nanocrystals, or nanolipids often introduced in a gel) have also shown promising results for encapsulation and enhanced skin delivery of actives [1,3,4,5,6]. However, these emerging technologies are inherently complex to develop and can have drug loading limitations that might restrict their adoption. Multiple water-in-oil-in-water emulsion was also reported as enhancing skin permeability but required a two-step emulsification procedure [7], increasing complexity and lengthening industrial production time.

There exist alternatives to conventional topical dosage forms that do not require complex manufacturing equipment or formulation processes. Cream–gel (CG) [8] is an oil-in-water system where the lipophilic phase is stabilised by a gel polymer network, not requiring the addition of surfactant, unlike emulgels. This kind of dosage form has recently gained exposure with the launch of a new retinoid for acne [9]. On the other hand, gel-in-oil (GIO) emulsions comprise a large volume of internal aqueous gel phase within a continuous lipophilic phase [10]. Both dosage forms have a relatively straightforward formulation process, with emulsification being possible even with low to medium shear agitation at room temperature [8,11,12]. There appear to be limited data available for the impact of these dosage forms on skin penetration of actives. Previous human skin experiments with cream–gel only have demonstrated superior API penetration, although the results are limited to one formulation with high variability and no characterisation of active content in skin compartments [13]. Our study is an attempt to bridge this gap, evaluating API penetration into and across human skin samples from both dosage forms with various excipient compositions.

Topical administration of diclofenac, a non-steroidal anti-inflammatory drug, enables localised treatment while limiting systemic exposure linked to gastrointestinal side-effects when compared to oral treatment. While the exact mechanism of diffusion through skin layers to the underlying layers has not yet been elucidated, it has been shown that muscular tissue concentration of diclofenac after topical applications in rats is not directly correlated to systemic concentration but mostly originates from direct skin diffusion [14]. Further microdialysis studies in human volunteers have shown that increased systemic concentration of diclofenac after cutaneous application was not linked to higher active content in subcutaneous adipose tissues or skeletal muscles, with lower concentration being observed for several treatments evaluated [15]. Based on these results, our target for a suitable diclofenac penetration profile for in vitro penetration testing (IVPT) was to maximise the API content in the skin sublayers (stratum corneum, epidermis, and dermis) while having similar or less diffusion in the fluid receptor, modelling systemic exposure, when compared to a market benchmark.

**Figure 1:**
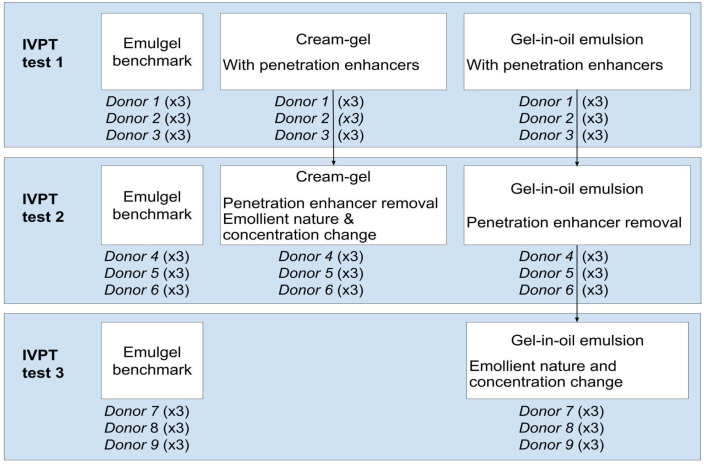
IVPT and formulation strategy.

To achieve this goal, the study was conducted step by step. Starting from cream–gel and gel-in-oil emulsion formulations with well-known penetration enhancers, compositions were then adjusted based on the first IVPT results to further investigate the impact of removing the penetration enhancers and adjusting the oily phase. The testing plan is described in Figure 1.

## 2. Results

### 2.1. Influence of Formulation Changes on Physical Stability

Cream–gel and gel-in-oil emulsion were initially formulated with well-known penetration enhancers to ensure sufficient penetration of diclofenac into the skin (Table 1). Propylene glycol (PG) and diethylene glycol monoethyl ether (DEGEE) were chosen. The composition of the benchmark product includes propylene glycol [16]. The second glycol was selected for its reported ability to adjust the skin delivery of active pharmaceutical ingredients [17]. The use of isopropyl myristate (IPM) as an emollient has also been demonstrated to increase penetration of diclofenac, in synergy with propylene glycol [18].

Formulation trials were carried out successfully; only gel-in-oil emulsion with both propylene glycol and diethylene glycol monoethyl ether showed physical instability during 45 °C follow-up (full stability data can be found in Supplementary Table S1).

Further formulation trials were carried-out, removing solvents and changing emollient, in order to obtain a penetration profile closer to that of the benchmark product. Isopropyl myristate was replaced by coco-caprylate/caprate (CCC) alone or combined with liquid paraffin, both being part of the benchmark composition. Medium-chain triglycerides (MCTs) were introduced into gel-in-oil emulsion as a common emollient in topical product development (Table 2 and Table 3).

All cream–gel prototypes were physically stable, whereas removing propylene glycol and increasing emollient concentration in gel-in-oil emulsions led to instability (full stability data can be found in Supplementary Tables S2 and S3).

### 2.2. Experimental Results of IVPT

In vitro penetration studies were carried out using Franz diffusion cells with human skin from abdominal surgery. Each test was performed on three different donors, with three replicates by donor (*n* = 9). The three donors are different between test 1, test 2, and test 3 (Figure 1). Skin samples were dermatomed to a thickness between 400 and 600 µm. Then, 10 mg/cm^2^ of the formulations were applied onto the skin. Penetration of diclofenac in the receptor fluid was assessed over a 24 h period, with the system temperature maintained at 34 °C. At the end of the experiment, stratum corneum was collected by tape-stripping and active content was quantified in the separate skin layers.

#### 2.2.1. Test 1: Starting Compositions with Glycols as Penetration Enhancer

In vitro penetration studies were carried out with formulations containing 10% propylene glycol alone or in combination with 20% diethylene glycol monoethyl ether, in comparison to the market benchmark.

**Figure 2:**
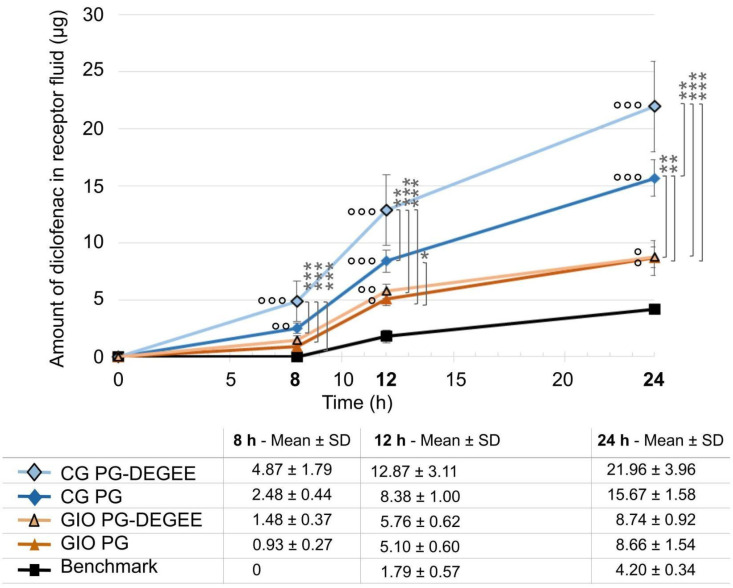
In vitro penetration profiles of cream–gel and gel-in-oil emulsion with PG or PG-DEGEE compared to benchmark. Amount of diclofenac penetrated into the receptor solution was expressed as mean ± SD (*n* = 9), with ° *p* < 0.05, °° *p* < 0.01, and °°° *p* < 0.001 for the statistical significance versus benchmark for a time point and * *p* < 0.05, ** *p* < 0.01, and *** *p* < 0.001 for the statistical significance between other formulations for a time point.

Prototypes tested show significantly higher penetration than benchmark in receptor solution from the 12 h time point (Figure 2). The percentages of diclofenac that penetrated in the receptor fluid at 24 h were 4.13% for the benchmark; 21.07% for the cream–gel combining DEGEE and propylene glycol (CG PG-DEGEE) and 15.00% for the cream–gel with propylene glycol alone (CG PG); 8.38% for the gel-in-oil emulsion combining DEGEE and propylene glycol (GIO PG-DEGEE) and 8.66% for the gel-in-oil emulsion containing only propylene glycol (GIO PG). Addition of DEGEE to cream–gel resulted in higher penetration of diclofenac from 8 h (*p* < 0.01 compared to trial with propylene glycol); however, no significant difference was seen between gel-in-oil emulsions (GIO).

#### 2.2.2. Test 2: Impact of Solvent Removal and Emollient Changes in Cream–Gel

Propylene glycol and diethylene glycol monoethyl ether were removed from formulations to obtain a penetration profile in receptor solution closer to the benchmark.

**Figure 3:**
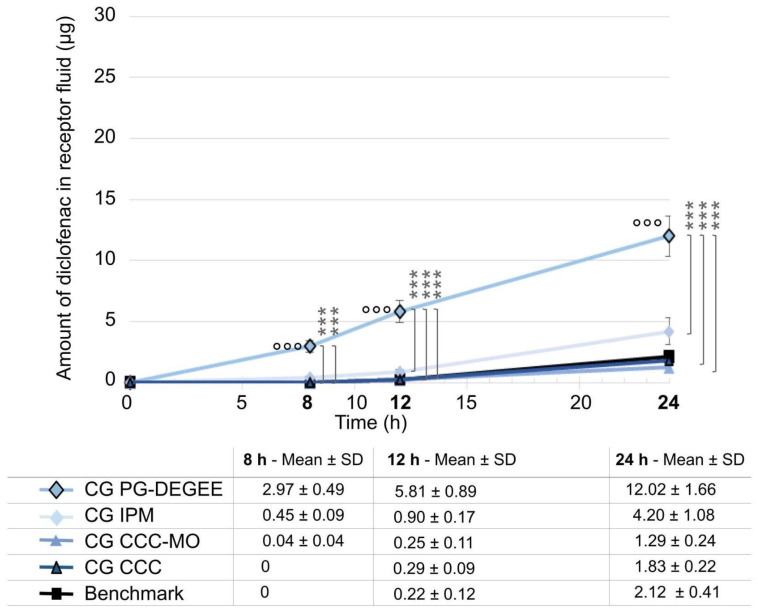
Comparison of cream–gel with solvent removal (PG-DEGEE versus without) and emollient changes to benchmark. Amount of diclofenac penetrated into the receptor solution was expressed as mean ± SD (*n* = 9), with °°° *p* < 0.001 for the statistical significance versus benchmark for a time point and *** *p* < 0.001 for the statistical significance between other formulations for a time point.

All three prototypes without solvents exhibited significantly lower diclofenac penetration (*p* < 0.001 compared to the formulation containing the two penetration enhancers), with no statistical difference to the benchmark (Figure 3). The percentages of diclofenac that penetrated in the receptor fluid at 24 h were 10.38% for the cream–gel with penetration enhancers (CG PG-DEGEE) compared to 3.75% for the cream–gel without penetration enhancers and containing the same emollient (isopropyl myristate: CG IPM); 1.15% for the cream–gel containing a combination of coco-caprylate/caprate and liquid paraffin (CG CCC-MO); 1.63% for the cream–gel containing coco-caprylate/caprate alone (CG CCC); 1.83% for the benchmark. Replacing 16% isopropyl myristate by 8% coco-caprylate/caprate alone or in association with 8% mineral oil results in lower diffusion in receptor solution, albeit not significantly lower.

**Figure 4:**
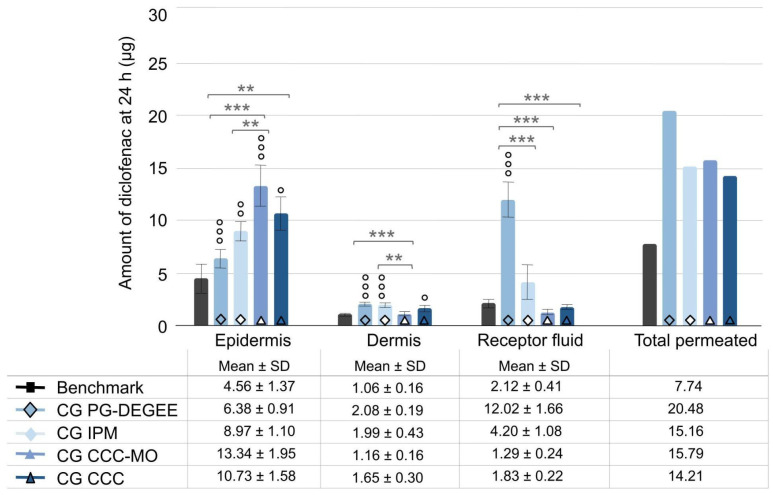
Comparison of cream–gel with solvent removal (PG-DEGEE versus without) and emollient changes to benchmark. Amount of diclofenac penetrated into skin layers and receptor fluid after 24 h was expressed as mean ± SD (*n* = 9), with ° *p* < 0.05, °° *p* < 0.01, and °°° *p* < 0.001 for the statistical significance versus benchmark for a time point and ** *p* < 0.01 and *** *p* < 0.001 for the statistical significance between other formulations.

Penetration of diclofenac into cutaneous layers at the end of the 24 h experiment was characterised. The three cream–gels without solvent show significantly higher diclofenac content in the epidermis compared to the benchmark, in addition to higher dermis content for CG IPM and CG CCC (Figure 4).

#### 2.2.3. Test 2: Impact of Solvent Removal in Gel-in-Oil Emulsion

**Figure 5:**
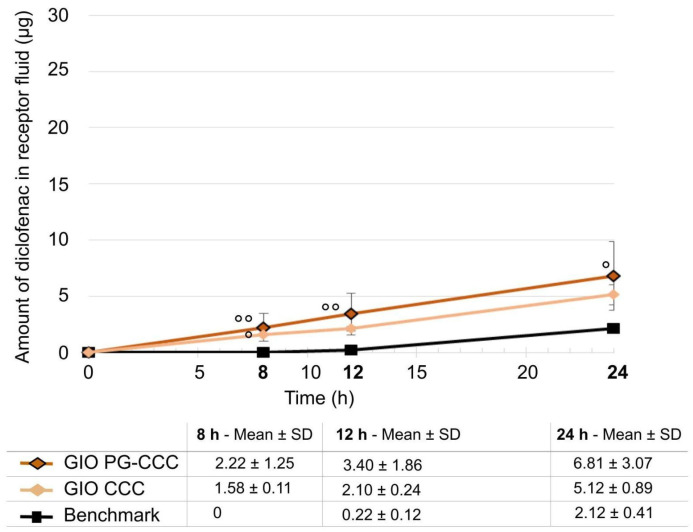
Comparison of gel-in-oil emulsion with solvent removal (PG versus without) to benchmark. Amount penetrated into the receptor solution was expressed as mean ± SD (*n* = 9), with ° *p* < 0.05, °° *p* < 0.01 for the statistical significance versus benchmark for a time point.

Gel-in-oil emulsion with 10% propylene glycol shows significantly increased diclofenac penetration in the receptor solution as compared to the benchmark (Figure 5; *p* < 0.01 at 8 and 12 h; *p* < 0.05 at 24 h). The percentages of diclofenac that penetrated in the receptor fluid at 24 h were 5.93% for the gel-in-oil emulsion with propylene glycol (GIO PG-CCC) and 4.42% for the formulation without propylene glycol compared to 1.83% for the benchmark. Removing propylene glycol leads to penetration kinetics closer to the benchmark, with only diclofenac content at 8 h revealing a statistical difference (*p* < 0.05)

**Figure 6:**
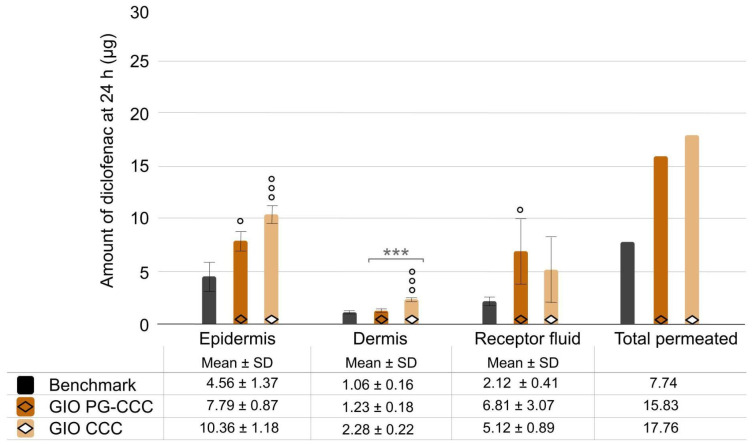
Comparison of gel-in-oil emulsion with solvent removal (PG-CCC versus CCC alone). Amount of diclofenac penetrated into skin layer and receptor fluid after 24 h was expressed as mean ± SD (*n* = 9), with ° *p* < 0.05, and °°° *p* < 0.001 for the statistical significance versus benchmark for a time point and *** *p* < 0.001 for the statistical significance between other formulations.

Removing propylene glycol from gel-in-oil emulsion did not change the total amount of diclofenac penetrated (Figure 6); it only changed skin distribution of the active, with significantly higher epidermis and dermis content versus the benchmark (*p* < 0.001 for both) and higher dermis content versus gel-in-oil emulsion with propylene glycol (*p* < 0.001).

#### 2.2.4. Test 3: Impact of Emollient Type and Concentration Changes in Gel-in-Oil Emulsion

**Figure 7:**
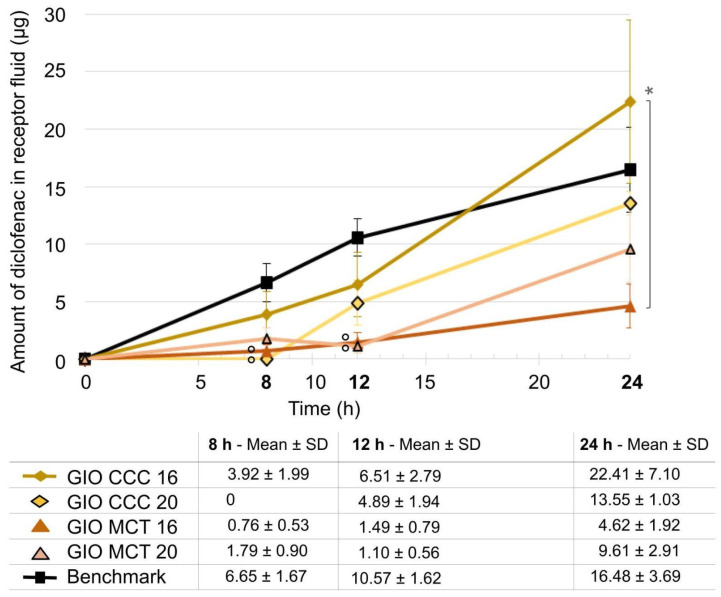
Comparison of gel-in-oil emulsion with changes in emollient type (CCC versus MCT) and concentration (16% versus 20%) to benchmark. Amount of diclofenac penetrated in the receptor solution was expressed as mean ± SD (*n* = 9), with ° *p* < 0.05 for the statistical significance versus benchmark for a time point and * *p* < 0.05 for the statistical significance between other formulations for a time point.

Increasing coco-caprylate/caprate concentration in gel-in-oil emulsion and replacement by medium-chain triglycerides (Figure 7) resulted in reduced penetration of diclofenac into the receptor fluid compared to the benchmark (significant at 8 h for 20% coco-caprylate/caprate, 8 and 12 h for 16% medium-chain triglycerides, and 12 h for 20% medium-chain triglycerides, with *p* < 0.05 for all). Only the gel-in-oil emulsion with 16% coco-caprylate/caprate had a similar penetration profile to the benchmark. The percentages of diclofenac that penetrated in the receptor fluid at 24 h were 17.03% for the gel-in-oil emulsion with 16% coco-caprylate/caprate (GIO CCC 16) compared to 12.80% for the benchmark; 10.44% for the formulation with 20% coco-caprylate/caprate; 3.50% and 7.15% for the gel-in-oil emulsions containing medium-chain triglycerides, 16% (GIO MCT 16) and 20% (GIO MCT 20), respectively.

Penetration of diclofenac into cutaneous layers at the end of the 24 h experiment was characterised, with further separation of the stratum corneum from the epidermis by tape-stripping for a more precise investigation.

**Figure 8:**
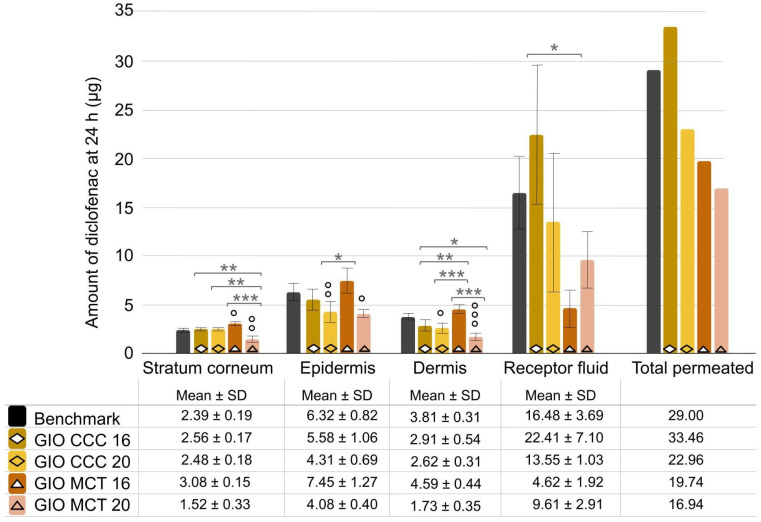
Comparison of gel-in-oil emulsion with changes in emollient type (CCC versus MCT) and concentration (16% versus 20%) to benchmark. Amount of diclofenac penetrated into skin layer and receptor fluid after 24 h was expressed as mean ± SD (*n* = 9), with ° *p* < 0.05, °° *p* < 0.01, and °°° *p* < 0.001 for the statistical significance versus benchmark for a time point and * *p* < 0.05, ** *p* < 0.01, and *** *p* < 0.001 for the statistical significance between other formulations.

Both gel-in-oil emulsions with 20% emollient resulted in significantly less diclofenac content in the epidermis and dermis as compared to the benchmark, while gel-in-oil emulsion with 16% coco-caprylate/caprate had a similar tissue distribution (Figure 8). Only the gel-in-oil formulation with 16% medium-chain triglycerides shows a superior tissue retention, with significantly higher diclofenac content in the stratum corneum versus the benchmark and in the dermis versus other gel-in-oil prototypes.

### 2.3. Rheological Characterisation

Further characterisation was carried out on representative and physically stable formulations of cream–gel (CG CCC) and gel-in-oil emulsion (GIO MCT 16) at Month 3 of ambient temperature stability monitoring (exploratory single measurement). 

**Figure 9:**
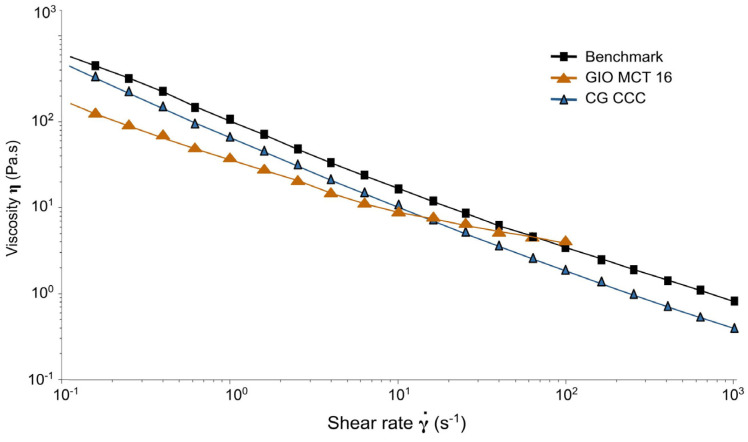
Flow curves of cream–gel (CG CCC), gel-in-oil emulsion (GIO MCT 16), and emulgel (benchmark) at Month 3 of storage at room temperature (single measurement). Only data below 100 s^−1^ shear rate are shown for GIO MCT 16, superior shear rates resulting in product ejection.

Interestingly, the cream–gel formulation showed a shear-thinning behaviour very close to the benchmark highlighted in Figure 9 by close viscosity at very low shear rates and similar slope of the curve and by close values of rate index (0.449 and 0.402, respectively, in Table 4). Oscillatory experiments also indicated a similar elastic structure at rest, as indicated by similar tan δ values in Table 4 (0.11 for the benchmark and 0.13 for the cream–gel), with slightly better shear recovery after stress for the cream–gel (shorter time and higher recovery rate; thixotropy results; Table 4). The gel-in-oil emulsion had an overall thinner consistency but displayed more Newtonian properties, visualised in Figure 9 by a lower slope of the curve and indicated by a rate index closer to 1 (Table 4). Oscillatory experiments highlighted a weaker structure, signalled both by a lower value of the complex modulus and a higher tan δ value (0.38 versus 0.11 and 0.13, respectively, for benchmark and cream–gel; Table 4). The gel-in-oil emulsion was also more prone to deformation, as exit from the linear domain into the plastic domain occurs at a lower oscillation stress σL (Table 4). As they were performed only once, after storage, these results represent only a first orientation and will have to be duplicated for confirmation.

## 3. Discussion

Use of human skin for in vitro penetration testing is generally recognised as the standard for predicting the penetration behaviour of the API from the formulation into the skin in clinical trials (OECD guidelines for the Testing of Chemicals, Section 4, Test No. 428 [19]). However, it is also known for its inherent lack of reproducibility, with high intra- and extra-donor variability [20,21,22]. This is clearly demonstrated by comparing the results given for the market benchmark formulation as, despite inclusion of several donors and replicates, diclofenac amount in the receptor fluid after 24 h in test 3 is more than three times higher than in test 1 and five times higher than in test 2. To minimise the impact of this variability on our conclusions, the impact of dosage form and formulation changes on diclofenac penetration was evaluated within the same test, with no data comparison being made from one test series to another. The benchmark was also included in all test series.

A penetration profile of diclofenac into the receptor fluid similar to the benchmark was obtained for cream–gel and gel-in-oil emulsions through formulation adjustments. While gel-in-oil emulsion with 16% coco-caprylate/caprate (GIO CCC 16) led to a matching diclofenac penetration into both receptor fluid and skin sub-layers, all cream–gels without permeation enhancers (CG IPM, CG CCC-MO, CG CCC) have shown significantly higher diclofenac penetration into skin layers compared to the benchmark. Superior penetration from CG IPM could arguably be attributed to the use of isopropyl myristate, an emollient with known permeation enhancing properties [18]. However, the choice of emollient cannot be the sole explanation, as cream–gels containing the same emollients as the benchmark (coco-caprylate/caprate, CG CCC; coco-caprylate/caprate and liquid paraffin, CG CCC-MO) still exhibit a superior diclofenac penetration in the skin layers. The possible influence of the concentration of different emollients cannot be excluded since the composition of the benchmark is only qualitatively known. Rheological analysis has evidenced close flow and oscillatory profiles between cream–gel and emulgel dosage forms, unlikely to explain differences in diclofenac skin penetration observed between these dosage forms. It is hypothesised that enhanced diclofenac penetration from the cream–gel could be linked to there being no surfactant added to cream–gel as compared to emulgel, resulting in a different thermodynamic equilibrium of the active within the formulation. In emulgels, the surfactant concentration varies from 1% to 10% and is in most cases between 1% and 5% [23,24,25,26,27,28,29]. In the cream–gel, a trace amount of surfactant comes only from the AMPS-based polymer, but its concentration in the cream gel remains below 0.4%. Further investigations by in vitro release testing could help verify this hypothesis. Another possibility is that cream–gel undergoes specific metamorphosis events after application on the skin, leading to higher diclofenac diffusion [30]. The polymeric network obtained at the core of cream–gels formulated in this study is based on electrostatic repulsion between anionic chains. The polymer, obtained by inverse polymerisation technology, includes AMPS monomer (i.e., acrylamido-2-methylpropane sulfonic acid). During production, the polymer is pre-neutralised using sodium hydroxide to obtain a ready-to use liquid ingredient (polymerisation occurs inside the water droplets of a water-in-oil emulsion). This pre-neutralisation step induces ionisation with negative charges borne by sulfonate functions. When introduced into an aqueous phase, the polymer chains expand spontaneously under the effect of the electrostatic repulsions between negative charges of sulfonate groups, generating a swelling of the polymers and the desired thickening effect [31]. Upon topical application, electrolytes on the skin surface are known to disrupt these interactions, leading to loss of viscosity. This phenomenon could lead directly to higher diclofenac diffusion from the dosage form into the skin, or indirectly by faster water evaporation, resulting in increased diclofenac flux. Through non-invasive methods such as confocal Raman spectroscopy, the impact of cream–gel on diclofenac diffusion from the dosage form into the different skin layers could be further studied.

Higher diclofenac penetration into skin layers from cream–gel formulations without solvent is even more surprising, given that the benchmark contains propylene glycol as a permeation enhancer and isopropyl alcohol that, upon evaporation, results in higher API flux. Using solvents is a well-known strategy to achieve higher API diffusion into the skin, although some solvents like propylene glycol have been shown to carry a risk of skin irritancy, especially when used at high concentration [32]. As such, safer alternatives for promoting skin penetration of APIs are highly desirable. Further experiments confirming the positive impact of cream–gel on API penetration would go a long way towards that goal.

In our experiment, only gel-in-oil formulas (GIO MCT 16, GIO MCT 20) have shown significantly lower diclofenac penetration compared to the benchmark. In the same way, gel-in-oil emulsions with the same solvent composition as cream–gels (GIO PG-DEGEE/CG PG-DEGEE, GIO PG/CG PG) demonstrated significantly reduced diffusion of diclofenac into the receptor fluid after 24 h. This can be explained by considering that, in gel-in-oil emulsion, diclofenac is predominantly located in the internal phase, as opposed to cream–gel and emulgel, where the aqueous phase is external. Thus, in gel-in-oil forms, diclofenac has to undergo an additional diffusion step from the aqueous phase to the external lipophilic phase before penetrating into the skin, which could explain its lower diffusion into the receptor fluid. Further investigations are needed with analysis time points closer to the beginning of IVPT experiments to confirm the existence of an increased lag-time. These findings show the potential of gel-in-oil emulsions for restricting systemic exposure to hydrophilic molecules as well as for sustained release.

Interestingly, compositional changes in our study did not induce the same impact in gel-in-oil emulsion or in cream–gel. From a physical stability perspective, cream–gels have shown more flexibility than gel-in-oil emulsions. From a solvent composition perspective, whereas removing diethylene glycol monoethyl ether from gel-in-oil emulsion did not change penetration into receptor fluid, cream–gel without DEGEE showed significantly reduced diffusion of diclofenac. In the same way, removing propylene glycol from gel-in-oil emulsion only affected the diclofenac distribution in the skin but not the total amount of diclofenac penetrated. This can once again be explained by the solvents being in the internal phase in gel-in-oil emulsions, potentially limiting their penetration enhancement effect.

Rheological characterisation has shown the similarity of cream–gel and emulgel, with only a slight structural loss after shear for the emulgel, which could be linked to either its polymer or surfactant composition. Their low rate index, indicative of a shear-thinning behaviour, and low viscosity, in the shear range corresponding to that applied when spreading on the skin (estimated to be between 500 s^−1^ and 10,000 s^−1^, depending on application velocity and film thickness [33,34]), favours the perception of a drug product easily spread on the skin [35,36]. On the other hand, the penetration-enhancing effect of massage has been demonstrated for anti-inflammatory drugs like diclofenac (acceleration of initial diffusion through the skin up to 8 h induced by 45 s of rubbing movement was reported [37]). From practitioner experience, maintaining a certain product presence/film thickness between the fingers and the skin facilitates glide and ensures an easy massage movement. Oils, displaying a Newtonian profile, are traditionally used for massage therapy due to their long playtime and lubricant properties [38,39]. The intermediate, more Newtonian, flowing profile of the gel-in-oil emulsion would help to prolong the playtime while ensuring sufficient skin penetration [40]. Further tribology assessment could be an interesting avenue to investigate the lubricating effect of gel-in-oil emulsion compared to a benchmark and support this assumption.

Studying the compatibility of these two dosage forms with medical devices is another potential avenue, such as the use of heat [41], oxygen flow [42], and an ultrasound-assisted technique (phonophoresis) [43], all reported to enhance the penetration of diclofenac.

Cream–gel and gel-in-oil emulsion have demonstrated differentiating diclofenac penetration profiles from conventional dosage forms. However, diffusion is also intrinsically linked to the physicochemical properties of an API, notably its partition coefficient and molecular weight [1]. Additional experiments should be carried out using APIs with different chemical structures to confirm the advantage of these dosage forms.

## 4. Materials and Methods

### 4.1. Chemicals

The benchmark product (Voltarenactigo 1%) was purchased from a local pharmacy. Diclofenac sodium was purchased from “Axo Industry International SA, Wavre, Belgium”. Propylene glycol (penetration enhancer) was purchased from “Brenntag S.A., Chassieu, France”. Diethylene glycol monoethyl ether (Transcutol^®^ P; penetration enhancer) was a kind gift from “Gattefossé, Saint-Priest, France”. Isopropyl myristate (DUB IPM; emollient) and medium-chain triglycerides (caprylic/capric triglyceride: DUB MCT 5545; emollient) were purchased from “Stéarinerie Dubois, Boulogne-Billancourt, France”. Liquid paraffin (Primol^®^ 352; emollient) was purchased from “Esso, Courbevoie, France”. AMPS-based polymer (Sepineo™ P 600: acrylamide/sodium acryloyldimetyltaurate copolymer and isohexadecane and polysorbate 80), cetearyl alcohol/cetearyl glucoside (Sepineo™ SE 68; surfactant), PEG-30 dipolyhydroxystearate (surfactant), coco-caprylate/caprate (emollient), and preservatives (Sepicide™ HB: phenoxyethanol and methylparaben and ethylparaben and propylparaben and butylparaben) were directly sourced from “Seppic SA, La Garenne Colombes, France”. Purified water was obtained through filtration by Elix^®^ Advantage 3 from “Merck KGaA, Darmstadt, Germany”.

### 4.2. Benchmark Composition

The packaging provides only qualitative information about the composition of the benchmark. It contains 1.16% of the active substance diclofenac diethylammonium, which corresponds to 1% diclofenac sodium, diethylamine, carbomer 974 (thickening polymer), cetomacrogol 1000 (surfactant), coco-caprylate/caprate (emollient), isopropyl alcohol (penetration enhancer), liquid paraffin (emollient), perfume creme 45 (containing benzyl benzoate and benzyl alcohol as preservatives), propylene glycol (penetration enhancer), and purified water [16].

### 4.3. Preparation of Formulations

**Figure 10:**
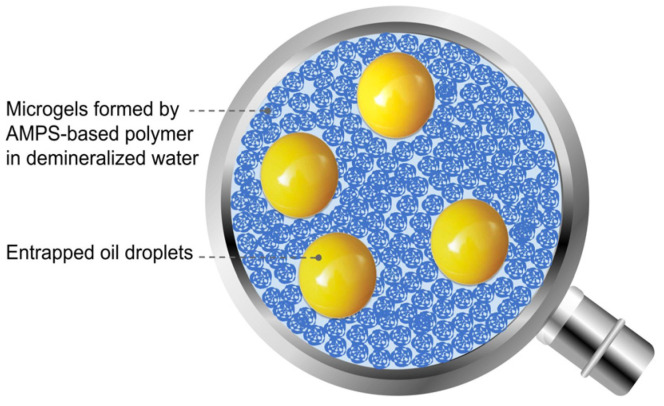
Schematic representation of a cream–gel microstructure.

Cream–gel is defined as the entrapment of a lipophilic phase in a hydrophilic gel without addition of surfactant [44] (Figure 10). Active phase was prepared by dissolution of diclofenac sodium in purified water at 50 °C under magnetic stirring. AMPS-based polymer was dispersed in the emollient (emollient or emollient combination used differs between the tests; see Table 1 and Table 2). Active phase was added on top of the lipophilic phase, then mixed under serrated disc agitation at a speed of 1000 rpm. Preservatives were added when a homogeneous cream–gel was obtained, confirmed by a smooth white, more or less opaque appearance. Alternatively, for cream–gel containing solvents, AMPS-based polymer was added to purified water and mixed under serrated disc agitation at a speed of 1000 rpm until a homogeneous gel was obtained. Diclofenac sodium was dissolved in the solvents before addition to the gel. Emollient was further added while maintaining agitation. Preservatives were added once a homogeneous cream–gel had been obtained.

**Figure 11:**
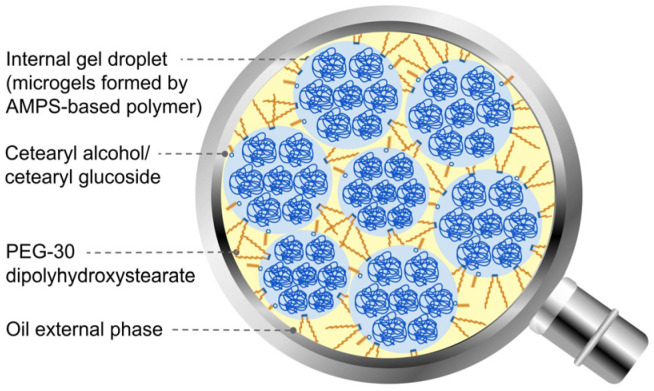
Schematic representation of a gel-in-in oil emulsion microstructure.

Gel-in-oil emulsion or high internal phase emulsions consist of a high concentration of closely packed gel droplets dispersed within a liquid continuous oil phase (Figure 11) [45]. Active phase was prepared by dissolving diclofenac sodium in part of the purified water at 50 °C or in the solvent system under magnetic stirring. Emulsifiers and emollients were melted together at 80 °C, then allowed to cool to room temperature before preservatives were added (emollient or emollient combination used differs between the tests; see Table 1, Table 2 and Table 3). AMPS-based polymer was added to purified water and mixed under serrated disc agitation at a speed of 1000 rpm until a homogeneous gel was obtained. Active phase was then added while maintaining agitation. Lipophilic phase was poured onto the gel and mixed with an anchor device for 1 min at 350 rpm followed by 10 min at 75 rpm.

### 4.4. Physical Stability Assessment

All formulations were assessed for physical stability over a 3-month period, at both room temperature 20 ± 2 °C (RT) in a closed environment away from light and 45 ± 5 °C (natural moisture levels). Formulations at 45 °C were stored in a lab oven UF750plus (Memmert GmbH + Co.KG, Schwabach, Germany). Visual appearance was assessed at Day 1, Day 7, Month 1, and Month 3 after manufacturing on samples kept at room temperature (RT) and at Day 7, Month 1, and Month 3 on samples stored at 45 °C. For cream–gels (with water continuous phase) pH was measured using a pH metre SevenEasy (Metler-Toledo, Greifensee, Switzerland) at Day 1, Day 7, Month 1, and Month 3 on samples kept at room temperature. For gel-in-oil emulsions, the presence of an oily continuous phase has been verified by a conductivity value ≤ 0.2 µS/cm (threshold defined as the upper limit for a water-in-oil emulsion). A previous study revealed that the evolution of conductivity was an early indicator of the risk of gel-in-oil emulsion destabilisation, which could result in the emulsion inversion [46]. The compliance with this threshold was confirmed using a conductometer pH/Cond 340i (WTW, Weilheim, Germany) at Day 7 and Month 1 on samples kept at room temperature. Viscosity was measured using a Brookfield LV viscometer (spindle 2 to 4, speed 6) for viscosities below 100,000 mPa·s and a Brookfield RV viscometer (spindle 7, speed 5) for viscosities above 100,000 mPa·s (Brookfield Engineering Laboratories Inc., Middleboro, MA, USA). Viscosity controls were carried out at Day 7 and Month 1 for samples kept at room temperature and at Month 1 after returning the sample to room temperature for samples stored at 45 °C (as long as the samples remain macroscopically homogeneous). Optical microscopy visualisation (×400 magnification; microscope Eclipse Ni-U, Nikon Europe B.V., Amstelveen, The Netherlands) was conducted on room temperature samples at Day 7, to assess diclofenac sodium crystallisation or lack thereof within the formulations. Measurements were performed once at each measurement point. The stability of a sample was determined by the absence of macroscopic signs of instability (heterogeneous appearance, exudation, sedimentation, phase separation), no significant variations in viscosity (greater than the standard deviation of the device ± 20% from the value at Day 7) or in pH (greater than the standard deviation of the apparatus ± 0.2), or a conductivity value below the threshold of 0.2 µS/cm.

Physicochemical parameters of the benchmark product were characterised when the primary packaging was opened, within the indicated shelf-life. As the product was purchased from a local pharmacy, the formulation manufacturing date could not be precisely determined. In addition, the quantity was too small to measure viscosity. Therefore, the only measurements taken before the IVPT tests were the visual appearance and pH.

### 4.5. IVPT Protocol

IVPT testing and diclofenac content analysis were performed externally at Biotoskin (Besançon, France). The study was approved by the Ministry of Higher Education, Research, and Innovation (Ethics Committee for research with human samples, CODECOH N°AC-2021-4468). In vitro penetration tests were carried out using Franz diffusion cells. For each test, human skin samples from three donors were obtained after abdominal surgery and after written informed consent of the donors, and were frozen at −20 °C. After thawing, skin samples were dermatomed to a thickness of 400 to 600 µm, then cut into disks. Receptor fluid (PBS + 0.1% sodium azide) was added into the experimental cells, and the skin samples deposited on top. The whole system was maintained at 34 °C using a water bath. After 1 h, transepidermal water loss was measured to check integrity of the skin samples. Formulations were applied onto the skin samples at a dose of 10 mg/cm^2^ using a finger cot (*n* = nine; three replicates per donor and three donors for each formulation, as shown in Figure 1).

Receptor fluid samples of 500 µL were taken and replaced at 8 h, 12 h, and 24 h after sample application. Samples were kept at 4 °C until analysis. After 24 h, the top of the skin samples was rinsed with 1 mL of a 50/50 water/ethanol solution. For test 3, stratum corneum was obtained by tape-stripping; the epidermis was mechanically separated from the dermis. Stratum corneum, epidermis, and dermis were put in tubes with 1 mL of extraction solvent (55% acetonitrile/45% phosphate buffer pH 3) and agitated for 24 h. After centrifugation at 4000 rpm, supernatants were collected and kept at 4 °C until analysis.

Diclofenac content was analysed using HPLC 1260 Infinity II (Agilent, Santa Clara, CA, USA) with UV diode array detector DAD HS (single measurement). An Ascentis C18 5 μm (250 × 4.6 mm) column was used as a stationary phase, with a pre-column Ascentis C18 5 μm (2 × 4 cm). Mobile phase, 55% acetonitrile and 45% phosphate buffer (10 mM) adjusted to pH 3 with acetic acid, was set at a flow rate of 0.8 mL/min. Diclofenac retention time was 14 min ± 1, with UV wavelength set at 277 nm. Linearity of diclofenac concentration was assessed from 0.049 to 100 μg/mL. The data are reported as mean ± standard deviation (SD) (n = 9). Two-Way Analysis of Variance (ANOVA) was carried-out, followed by a Fisher test if necessary. Statistical significance was based on a 95% confidence interval. The percentages of diclofenac that penetrated in the receptor fluid at 24 h was calculated as follows:

$$\frac{Diclofenac\;quantity\;in\;receptor\;fluid\;after\;24\;hours}{Quantity\;of\;formula\;applied\times theoretical\;concentration\;of\;Diclofenac\;within\;formula}$$

in which the quantity of formula applied was obtained by reverse weighing after application.

### 4.6. Rheological Characterisation

Rheology experiments were attempted on formulations with relevant diclofenac penetration. This first screening, carried out after three months of storage at room temperature, was intended to see whether the two forms, cream–gel and gel-in-oil emulsion, had behaviours that could be related to penetration profiles. Given the exploratory nature, only one measure was carried out on each experiment. Rheological testing was carried out using a rotational controlled stress/strain rheometer Discovery Hybrid Rheometer DHR-2^®^ (Waters—TA Instruments, New Castle, DE, USA), at controlled temperature of 20 °C and with a 40 mm 2.0° cone plate geometry with a smooth surface. Flow experiments were carried out using a flow sweep protocol with logarithmic sweep from 0.01 s^−1^ to 1000 s^−1^ shear rate, with 5 s equilibration time and 30 s averaging time. A scaled time average factor of 0.2 was used. Results were analysed using the Herschel Bulkley model. Thixotropy was evaluated using a 3-step flow peak hold protocol: 120 s at 0.01 s^−1^ shear rate, 60 s at 30 s^−1^ shear rate, and 300 s at 0.01 s^−1^ shear rate. Thixotropy time is defined as the time to recover 80% of the average viscosity during the third step, and structural recovery is the ratio between average viscosities at the first and third steps. Oscillation tests were performed using an amplitude sweep protocol at 1 Hz frequency with logarithmic sweep from 0.01% to 1000% strain.

## 5. Conclusions

Cream–gels and gel-in-oil emulsions were found as suitable vehicles to stabilise diclofenac, but the composition adjustments of the formula dictated different penetration profiles.

Cream–gel dosage form allows similar penetration into the receptor fluid and higher concentrations in the skin layers compared to the benchmark, without the need for penetration enhancers. Addition of penetration enhancers increased diclofenac levels in receptor fluid. This dosage form was also shown to be very flexible, as formulation changes had limited to no impact on the physical stability. 

For gel-in-oil emulsions, addition of polar penetration enhancer had a more limited impact on penetration of diclofenac in receptor fluid, whereas changing the nature of the emollient constituting the external phase of the emulsion led to a more profound impact on API penetration. Interestingly, only this dosage form led to significantly reduced amounts of diclofenac in receptor fluid compared to benchmark, opening the possibility for lower systemic exposure of APIs. Complementary studies, especially on other oil phase compositions, could be of interest to optimise the stability of the formulation.

For both dosage forms, further research will be essential to determine if the findings of this study can be applied to other APIs.

## 6. Patents

Seppic also holds a patent on the gel-in-oil dosage form for topical use comprising at least one anti-inflammatory substance. None of the authors are among the inventors.

## Figures and Tables

**Table 1:** Composition (% *w*/*w*) and physical stability of cream–gel and gel-in-oil emulsions with solvents for IVPT test 1.

Dosage Form		Emulgel	Cream–Gel	Cream–Gel	Gel-in-Oil	Gel-in-Oil
Chemical	Trial code	Benchmark [16]	CG PG	CG PG-DEGEE	GIO PG	GIO PG-DEGEE
Diclofenac sodium		1%	1%	1%	1%	1%
Propylene glycol		Unknown *	10%	10%	10%	10%
Diethylene glycol monoethyl ether		/	/	20%	/	20%
Isopropyl myristate		/	16%	16%	16%	16%
Coco-caprylate/caprate		Unknown *	/	/	/	/
Liquid paraffin		Unknown *	/	/	/	/
AMPS-based polymer		/	4%	4%	4%	4%
Cetearyl alcohol/cetearyl glucoside		/	/	/	1%	1%
PEG-30 dipolyhydroxystearate		/	/	/	3%	3%
Preservatives		/	1%	1%	1%	1%
Purified water		Up to 100%	Up to 100%	Up to 100%	Up to 100%	Up to 100%
Control at day 7 at RT (Room Temperature)	Visual inspection	Pourable, white **	Compact, white, shiny, smooth	Pourable, white, shiny, smooth	Pourable, white, shiny, smooth	Liquid, white, shiny, smooth
	Viscosity *** (mPa·s)	NA ****	60,000	55,000	64,000	5000
	pH	7.2 **	8.2	7.4	NA ****	NA****
	Conductivity (µS/cm)	NA ****	NA ****	NA ****	≤0.2	≤0.2
	API crystallisation	None **	None	None	None	None
Stability at 3 months at RT and 45 °C		NA ****	Stable	Stable	Viscosity loss at 1 month/No visual instability	Viscosity loss at 1 month at 45 °C/1% phase separation at 3 months at 45 °C

* The presence of the excipient in the benchmark is known from the package insert, but no information is available on its concentration. ** Physicochemical parameters of the benchmark product were characterised when the primary packaging was opened, within the indicated shelf-life. *** Brookfield LV viscometer with spindle 3 at speed 6 if viscosity < 20,000 mPa·s; with spindle 4 if >20,000 mPa·s. **** Not Applicable.

**Table 2:** Composition (% *w*/*w*) and physical stability of cream–gel and gel-in-oil emulsions when removing solvent and changing emollient for IVPT test 2.

Dosage Form		Emulgel	Cream–Gel	Cream–Gel	Cream–Gel	Cream–Gel	Gel-in-Oil	Gel-in-Oil
Chemical	Trial code	Benchmark	CG PG-DEGEE	CG IPM	CG CCC-MO	CG CCC	GIO PG-CCC	GIO CCC
Diclofenac sodium		1%	1%	1%	1%	1%	1%	1%
Propylene glycol		Unknown *	10%	/	/	/	10%	/
Diethylene glycol monoethyl ether		/	20%	/	/	/	/	/
Isopropyl myristate		/	16%	16%	/	/	/	/
Coco-caprylate/caprate		Unknown *	/	/	8%	8%	16%	16%
Liquid paraffin		Unknown *	/	/	8%	/	/	/
AMPS-based polymer		/	4%	4%	4%	4%	4%	4%
Cetearyl alcohol/cetearyl glucoside		/	/	/	/	/	1%	1%
PEG-30 dipolyhydroxystearate		/	/	/	/	/	3%	3%
Preservatives		/	1%	1%	1%	1%	1%	1%
Purified water		Up to 100%	Up to 100%	Up to 100%	Up to 100%	Up to 100%	Up to 100%	Up to 100%
Control at day 7 at RT	Visual inspection	Pourable, white **	Pourable, white, shiny, smooth	Pourable, white, smooth	Pourable, white, smooth	Compact, white, smooth	Liquid, white	Pourable, white
	Viscosity *** (mPa·s)	NA ****	55,000	65,000	64,000	57,000	14,490	56,300
	pH	7.2 **	7.4	8.8	8.3	8.2	NA ****	NA ****
	Conductivity (µS/cm)	NA ****	NA ****	NA ****	NA ****	NA ****	≤0.2	≤0.2
	API crystallisation	None **	None	None	None	None	None	None
Stability at 3 months at RT and 45 °C		NA ****	Stable	Stable	Stable	Stable	Viscosity loss at 1 month at 45 °C/1% phase separation at 3 months at 45 °C	Viscosity loss at 1 month at 45 °C/Beginning of phase separation at 3 months at 45 °C

* The presence of the excipient in the benchmark is known from the package insert, but no information is available on its concentration. ** Physicochemical parameters of the benchmark product were characterised when the primary packaging was opened, within the indicated shelf-life. *** Brookfield LV viscometer with spindle 3 at speed 6 if viscosity < 20,000 mPa·s; with spindle 4 if >20,000 mPa·s. **** Not Applicable.

**Table 3:** Composition (% *w*/*w*) and physical stability of gel-in-oil emulsions when changing emollient type and concentration (%*w*/*w*) for IVPT test 3.

Dosage Form		Emulgel	Gel-in-Oil	Gel-in-Oil	Gel-in-Oil	Gel-in-Oil
Chemical	Trial code	Benchmark	GIO CCC 16	GIO CCC 20	GIO MCT 16	GIO MCT 20
Diclofenac sodium		1%	1%	1%	1%	1%
Coco-caprylate/caprate		Unknown *	16%	20%	/	/
Medium-chain triglyceride		/	/	/	16%	20%
Coco-caprylate/caprate		Unknown *	/	/	/	/
Liquid paraffin		Unknown *	/	/	/	/
AMPS-based polymer		/	4%	4%	4%	4%
Cetearyl alcohol/cetearyl glucoside		/	1%	1%	1%	1%
PEG-30 dipolyhydroxystearate		/	3%	3%	3%	3%
Preservatives		/	1%	1%	1%	1%
Purified water		Up to 100%	Up to 100%	Up to 100%	Up to 100%	Up to 100%
Control at day 7 at RT	Visual inspection	Pourable, white **	Liquid, white, smooth	Liquid, white, smooth	Compact, white, smooth	Liquid, white, smooth
	Viscosity *** (mPa·s)	NA ****	14,300	7320	103,000	26,000
	pH	7.2 **	NA ****	NA ****	NA ****	NA ****
	Conductivity (µS/cm)	NA ****	≤0.2	≤0.2	≤0.2	≤0.2
	API crystallisation	None **	None	None	None	None
Stability at 3 months at RT and 45 °C		NA ****	Beginning of phase separation at 45 °C	1% phase separation at 7 days at 45 °C and 1 month at RT	Stable	Beginning of phase separation at 7 days at 45 °C

* The presence of the excipient in the benchmark is known from the package insert, but no information is available on its concentration. ** Physicochemical parameters of the benchmark product were characterised when the primary packaging was opened, within the indicated shelf-life. *** Brookfield LV viscometer with spindle 3 at speed 6 if viscosity < 20,000 mPa·s; with spindle 4 if >20,000 mPa·s; Brookfield RV viscometer with spindle 7 at speed 5 if >100,000 mPa·s. **** Not Applicable.

**Table 4:** Rheological characterisation results from flow, thixotropy, and oscillatory measurements at Month 3 of storage at room temperature (single measurement).

Trial Code			Benchmark	CG CCC	GIO MCT 16
Dosage form			Emulgel	Cream–gel	Gel-in-oil emulsion
Flow		Rate index n	0.449	0.402	0.708
		Yield stress (Pa)	30.8	19.4	11.3
Thixotropy		Recovery time (s)	11	7	28
		Structural recovery (%)	84	105	140
Oscillation Amplitude	Linear domain	Complex modulus G* (Pa)	368	388	184
		tan δ	0.11	0.13	0.38
	Plastic domain	σL (Pa)	5.64	10.79	2.24

## Data Availability

The data presented in this study are available on request from the corresponding author. The data are not publicly available due to confidentiality restrictions.

## Supplementary Materials

The supporting information can be downloaded at: https://www.mdpi.com/article/10.3390/ijms25137432/s1.

## Abbreviations

APMS	2-Acrylamido-2-Methyl-Propane-Sulfonic Acid
ANOVA	Analysis of Variance
API	Active Pharmaceutical Ingredient
CCC	Coco-caprylate/caprate
CG	Cream–gel
DEGEE	Diethylene glycol monoethyl ether
IPM	Isopropyl myristate
IVPT	In vitro Permeation Test
HPLC-UV	High-Performance Liquid Chromatography–Ultra-Violet spectroscopy
GIO	Gel-in-Oil
MCT	Medium-chain Triglyceride
MO	Mineral oil
NA	Not applicable
OECD	Organization for Economic Cooperation and Development
PBS	Phosphate buffer saline
PG	Propylene glycol
pH	Potential of Hydrogen References
RT	Room temperature
SD	Standard Deviation
