# Supplementary material for: Alternatives to Conventional Topical Dosage Forms for Targeted Skin Penetration of Diclofenac Sodium
Source: Int J Mol Sci. 2024 Jul 6;25(13):7432. doi: 10.3390/ijms25137432 (PMC11242280; doi:10.3390/ijms25137432)
Supplement: Supplementary file 1 [file ijms-25-07432-s001.zip › ijms-3048844-supplementary.pdf]

Table S1. Physical stability of cream-gels (Test IVPT 1 and 2)

Trial code		CG PG	CG PG-DEGEE	CG IPM	CG CCC-MO	CG CCC
**Control at day 1** at RT	Visual inspection	Compact, white, shiny, smooth	Pourable, white, shiny, smooth	Compact, white, smooth	Pourable, white, smooth	Compact, white, smooth
	pH	8.3	7.6	8.8	8	8.1
**Control at day 7** at RT	Visual inspection	Compact, white, shiny, smooth	Pourable, white, shiny, smooth	Compact, white, smooth	Pourable ,white, smooth	Compact, white, smooth
	pH	8.2	7.4	8.8	8.3	8.2
	Viscosity*(mPa.s)	60,000	55,000	65,000	64,000	57,000
	API crystallisation	None	None	None	None	None
at45°C	Visual inspection	Pourable, white, shiny, smooth	Pourable, white, shiny, smooth	Compact, white, smooth	Pourable, white, smooth	Pourable, white, smooth
**Control at Month 1** at RT	Visual inspection	Pourable, white, shiny, smooth	Pourable, white, shiny, smooth	Compact, white, smooth	Pourable, white, smooth	Pourable, white, smooth
	pH	8.1	7.4	8.5	7.9	8.0
	Viscosity*(mPa.s)	58,000	49,000	63,500	61,000	47,500
at45°C	Visual inspection	Pourable, white, shiny, smooth	Pourable, white, shiny, smooth	Pourable, white, smooth	Pourable, white, smooth	Pourable, white, smooth
	Viscosity*(mPa.s)	86,000	58,500	68,000	63,000	66,700
**Control at Month 3** at RT	Visual inspection	Compact, white, shiny, smooth	Pourable, white, shiny, smooth	Compact, white, smooth	Pourable, white, smooth	Pourable, white, smooth
	pH	8.2	7.3	8.5	7.9	7.9
at 45°C	Visual inspection	Compact, white, shiny, smooth	Pourable, white, shiny, smooth	Pourable, white/beige, smooth	Pourable, white, smooth	Pourable, white, smooth

*Brookfield LV viscometer with spindle 4.

Table S2. Physical stability of gel-in-oil emulsions (Test IVPT 1 and 2)

Trial code		GIO PG	GIO PG-DEGEE	GIO PG-CCC	GIO CCC
**Control at day 1** at RT	Visual inspection	Pourable, white, shiny, smooth	Liquid, white, shiny, smooth	Liquid, white	Pourable, white
	Conductivity (µS/cm)	≤ 0.2	≤ 0.2	≤ 0.2	≤ 0.2
**Control at day 7** at RT	Visual inspection	Pourable, white, shiny, smooth	Liquid, white, shiny, smooth	Liquid, white	Pourable, white
	Conductivity (µS/cm)	≤ 0.2	≤ 0.2	≤ 0.2	≤ 0.2
	Viscosity*(mPa.s)	64,000	5,000	14,490	56,300
	API crystallisation	None	None	None	None
at45°C	Visual inspection	Liquid, white, shiny, smooth	Liquid, white, shiny, smooth	Liquid, white	Liquid, white
**Control at Month 1** at RT	Visual inspection	Liquid, white, shiny, smooth	Liquid, white, shiny, smooth	Liquid, white	Pourable, white
	Conductivity (µS/cm)	≤ 0.2	≤ 0.2	≤ 0.2	≤ 0.2
	Viscosity*(mPa.s)	43,000	5,000	13,350	71,600
at45°C	Visual inspection	Liquid, white, shiny, smooth	Liquid, white, shiny, smooth	Liquid, white	Pourable, white
	Viscosity*(mPa.s)	39,000	2,700	6,880	24,000
**Control at Month 3** at RT	Visual inspection	Liquid, white, shiny, smooth	Liquid, white, shiny, smooth	Liquid, white	Pourable, white
at 45°C	Liquid, yellowish, smooth	Compact, white, shiny, smooth	1% phase separation	1% phase separation	Beginning of phase separation

* Brookfield LV viscometer with spindle 3 at speed 6 if viscosity < 20,000 mPa.s; with spindle 4 if > 20,000 mPa.s

Table S3. Physical stability of gel-in-oil emulsions (IVPT test 3).

Trial code		GIO CCC 16	GIO CCC 20	GIO MCT 16	GIO MCT 20
**Control at day 1** at RT	Visual inspection	Liquid, white, smooth	Liquid, white, smooth	Compact, white, smooth	Liquid, white, smooth
	Conductivity (µS/cm)	≤ 0.2	≤ 0.2	≤ 0.2	≤ 0.2
**Control at day 7** at RT	Visual inspection	Liquid, white, smooth	Liquid, white, smooth	Compact, white, smooth	Liquid, white, smooth
	Conductivity (µS/cm)	≤ 0.2	≤ 0.2	≤ 0.2	≤ 0.2
	Viscosity*(mPa.s)	14,300	7,320	103,000	26,000
	API crystallisation	None	None	None	None
at45°C	Visual inspection	Liquid, white, smooth	1% phase separation	Pourable, white, shiny	Liquid, white, beginning of phase separation
**Control at Month 1** at RT	Visual inspection	Liquid, white, shiny, smooth	1% phase separation	Compact, white, smooth	Liquid, white, smooth
	Conductivity (µS/cm)	≤ 0.2	*Not performed*	≤ 0.2	≤ 0.2
	Viscosity*(mPa.s)	11,000	*Not performed*	89,000	25,700
at45°C	Visual inspection	Liquid, white, smooth	5% phase separation	Pourable, white, smooth	1% phase separation
	Viscosity*(mPa.s)	14,100	*Not performed*	100,000	*Not performed*
**Control at Month 3** at RT	Visual inspection	Liquid, white, smooth	*Not performed*	Compact, white, smooth	Liquid, white, smooth
at 45°C	Liquid, yellowish, smooth	Beginning of phase separation	*Not performed*	Pourable, white, smooth	*Not performed*

* Brookfield LV viscometer with spindle 3 at speed 6 if viscosity < 20,000 mPa.s; with spindle 4 if > 20,000 mPa.s
